# First reported double drug–drug interaction in a cancer renal patient under everolimus treatment: therapeutic drug monitoring and review of literature

**DOI:** 10.1186/s40001-023-01172-w

**Published:** 2023-06-29

**Authors:** Eduard Fort-Casamartina, Carme Muñoz-Sanchez, Raul Francisco Rigo-Bonnin, Pamela Maria del Valle-Celiz, Núria Gonzalo-Diego, Sara Otero-Torres, Carmen Bleda-Perez, Judith Prats-Jimenez, Sandra Fontanals-Martínez

**Affiliations:** 1 Pharmacy Service, Hospital Duran and Reynals (Catalan Institute of Oncology), Gran Via de l’Hospitalet 199–203, Hospitalet de Llobregat, 08908 Barcelona, Spain; 2 Clinical Laboratory, University Bellvitge Hospital, Carrer de La Feixa Llarga S/N, Hospitalet de Llobregat, 08907 Barcelona, Spain; 3 Medical Oncology, Hospital Duran and Reynals (Catalan Institute of Oncology), Gran Via de l’Hospitalet 199–203, Hospitalet de Llobregat, 08908 Barcelona, Spain

**Keywords:** Everolimus, Pharmaceutical care, Therapeutic drug monitoring, Pharmacokinetic, Drug–drug interaction, CYP3A4 metabolism, Targeted drugs, Personalized treatment

## Abstract

Everolimus is an inhibitor of mammalian target of rapamycin (mTOR) used in both transplantation and cancer treatment (breast, renal and neuroendocrine). In transplantation, therapeutic drug monitoring (TDM) is recommended due to the potential drug–drug interactions with chronic medications, which can affect everolimus pharmacokinetics. In cancer treatment, everolimus is used at higher doses than in transplantation and without a systematic drug monitoring.

We present a case report of a 72-year-old woman with epilepsy history to whom everolimus 10 mg QD was prescribed as third line of treatment for renal cell carcinoma (RCC). The potential drug interactions between everolimus and the patient's chronic medications, carbamazepine and phenytoin, are significant as both are known as strong inducers CYP3A4 metabolism, potentially leading to underexposure to everolimus.

TDM of everolimus was recommended by the pharmacist. The literature suggests that a minimum plasma concentration (Cminss) of everolimus over 10 ng/ml is associated with better response to treatment and progression-free survival (PFS). The patient’s everolimus dose had to be increased until 10 mg BID, and regular monitoring of everolimus levels showed an increase in Cminss from 3.7 ng/ml to 10.8 ng/ml.

This case highlights the importance of checking for potential drug interactions and monitoring everolimus levels in patients on chronic medication, especially those with several inducers or inhibitors of CYP3A4 metabolism. TDM can help to ensure that patients are treated with their optimal dose, which can improve the effectiveness of the treatment or minimize the risk of toxicities.

## Background

RCC is the most common type of kidney cancer in adults, representing 3% of all cancers in women and 5% in men with an incidence of around 400.000 cases worldwide (30% of patients are metastatic at diagnosis) [1].

The first-line treatment options for metastatic RCC have improved significantly over the past 20 years, with the development of targeted therapies such as tyrosine kinase inhibitors (TKIs) and immune checkpoint inhibitors (ICI). In recent clinical trials, combination therapy with antiangiogenic drugs and ICI, or two ICI’s may represent a promising approach for first-line treatment of metastatic RCC [2–5]. The choice of subsequent lines of therapy depends on several factors, including the patient’s prior treatment history, performance status, and comorbidities [1]. In METEOR and RECORD-1 trial everolimus increase PFS in RCC when was used as second- or third-line therapy [6].

**Fig. 1:**
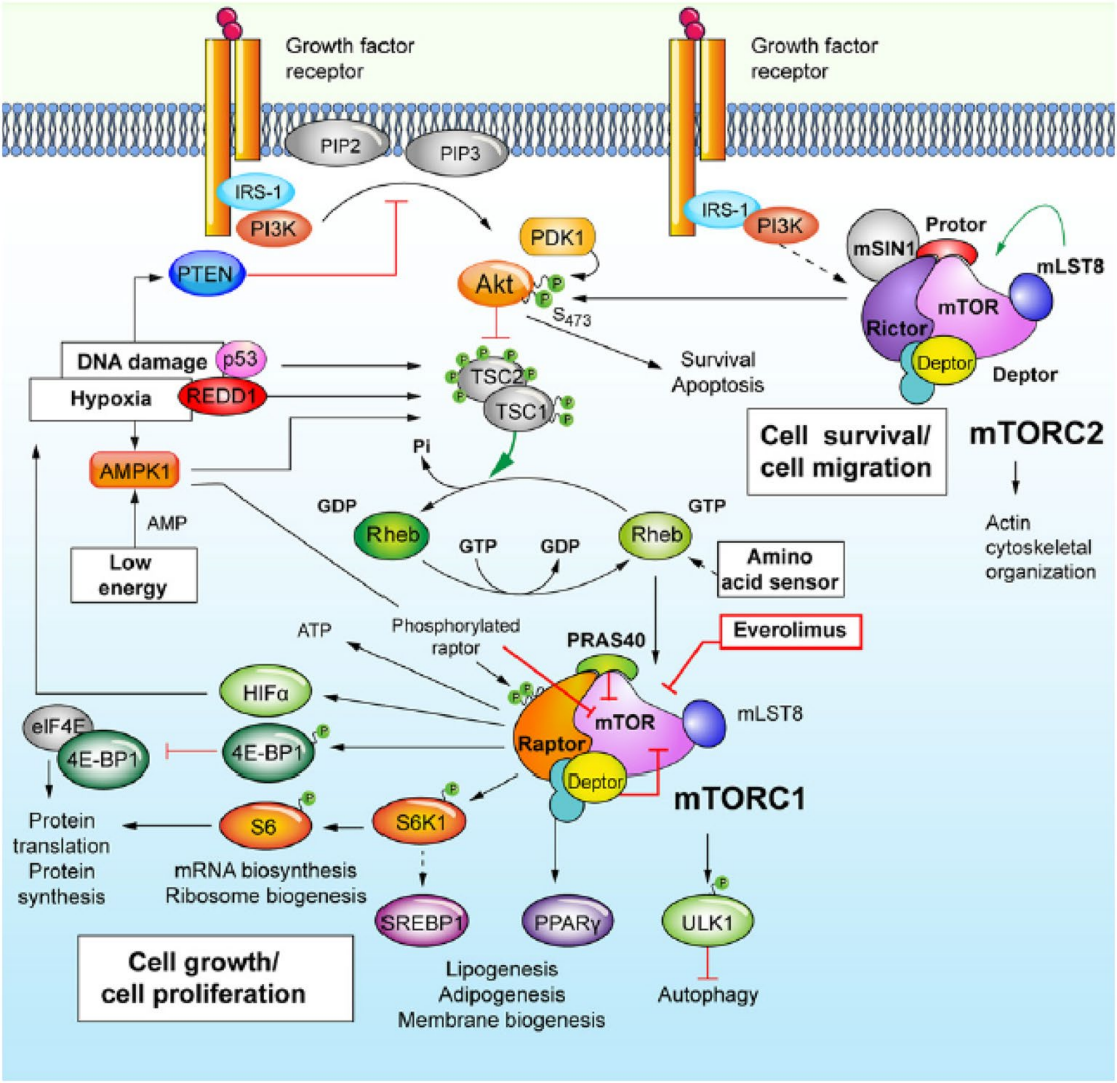
mTOR signaling pathway

Everolimus is an orally administered rapamycin derivate inhibiting the mammalian target of rapamycin (mTOR). This is a key signaling molecule in the phosphatidylinositol 3-kinase (PI3K)/Akt pathway which is involved in the regulation, growth, proliferation, metabolism, survival and angiogenesis of cells that is often dysregulated in cancer (Fig. 1). Nowadays it is used for cancer treatment at a fixed dose of 10 mg/daily for metastatic renal cell cancer and in neuroendocrine tumors, and in combination with exemestane for advanced hormone receptor positive (HR +), negative human epidermal growth factor-2 (HER2-) breast cancer. [7–9]

Everolimus is also used in transplant patients as immunosuppressant. Due to its narrow therapeutic index and pharmacokinetic inter-individual variability, routine TDM is recommended to maintain a Cminss between 3–8 ng/ml [10, 11]^.^ In cancer patients, Cminss below 10 ng/ml have been associated with worse response to treatment while Cminss higher than 26.3 ng/ml has been related to higher incidence of adverse events. However, in cancer setting, TDM is not currently performed. Variability in everolimus blood exposure may be influenced by several factors, including age, sex, body composition, genetic factors and drug–drug interactions which could affect its hepatic metabolism by cytochrome CYP3A4 [12–14].

We present the first double interaction with everolimus in a case report of a 72-year-old woman with a history of RCC and previous nephrectomy. Treatment with everolimus as third-line treatment was started. She was also taking carbamazepine and phenytoin for epilepsy, two majors inducers of CYP3A4 resulting in heavily decreased everolimus levels. This interaction was confirmed by TDM and everolimus dosage had to be increased, from 10 mg QD until 10 mg BID. Finally optimal Cminss was achieved.

## Case presentation

A 72-year-old woman with smoking and epilepsy history was diagnosed from a stage pT3a renal carcinoma in December 2015. Radical right nephrectomy was performed in February 2016. In September 2018, a right lung segmentectomy was practiced due to lepidic adenocarcinoma growth. On February 2021 progression was detected with right adrenal massive bleeding, hilar adenopathy and left renal adrenal metastasis. Nivolumab was initiated as first-line treatment in May 2021 for intermediate-risk clear-cell RCC. As adverse events dry mouth, grade II anorexia and grade I astheny were reported, with no immune-mediated toxicities. In December 2021, new progression was detected and second-line treatment with cabozantinib 60 mg daily was started. Enalapril was prescribed due to hypertension grade I (with no cabozantinib dose reduction), and patient also referred nausea and diarrhea grade I. A new progression was detected and third-line treatment with everolimus at 10 mg QD in fasted conditions (1 h before breakfast) was started in the 21st of March, 2022.

Patient chronic medication plan was checked by the hospital pharmacist: carbamazepine 400 mg BID, phenytoin 100 mg BID and acetaminophen 650 mg plus tramadol 75 mg TID. By using two databases (lexicomp and drugs), two drug interactions were detected: carbamazepine and phenytoin are classified as strong inducers of CYP3A4 metabolism and could lead to decrease everolimus Cminss.

This finding was discussed within the multidisciplinary team, including the oncologist and the pharmacist, and a TDM plan was planned. Method used for analysis was ultra-high-performance liquid chromatography coupled to tandem mass spectrometry. Four weeks after starting treatment, patient had no toxicities and first TDM result showed a Cminss of 3.7 ng/ml, which was considered to be an underexposure to everolimus.

**Fig. 2:**
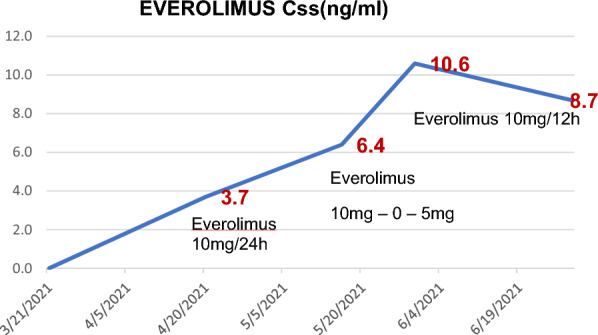
Everolimus dosage and Cminss evolution

Everolimus dose was increased from 10 mg QD to 15 mg daily (administered 10 mg, 1 h before breakfast and 5 mg, 1 h before dinner). Four weeks later TDM showed higher Cminss, from 3.7 to 6.4 ng/ml, without relevant toxicities. However, a second dose adjustment was needed, 10 mg BID, administered before meals. Two weeks and a month later, new TDM were planned and Cminss were 10.6 ng/ml and 8.7 ng/ml, respectively (Fig. 2). During everolimus treatment, stable disease was achieved, and no relevant toxicities were observed. Unfortunately, everolimus treatment was stopped on July 2022, due to lung progression, pleural effusion, and respiratory insufficiency, and finally patient died.

## Discussion

Everolimus is an oral mTOR inhibitor that binds with high affinity to the FK506 binding protein-12 (FKBP-12), and activation of mTOR is inhibited by this complex. Due to several factors could affect its pharmacokinetic profile, TDM is routinely established when everolimus is used in transplant patients to achieve the optimal Cmins, whereas in cancer patients is not established [15].

According to product label, in oncology, everolimus is prescribed as a standard fixed oral dose of 10 mg QD. It is absorbed rapidly and peak concentration is reached after 1.3–1.8 h. The systemic availability of a single oral 10 mg dose of everolimus is significantly reduced by co-administration with a meal compared with fasting conditions. Maximum concentration (Cmax) and area under the concentration–curve time (AUC) were reduced by 42% and 22% after low-fat meals; this reduction increased until 54% and 33% after a high-fat meal [16]. We recommended our patient to take everolimus every day in fasting conditions, at least 1 h before breakfast. Everolimus steady (SS) state is reached within 7–14 days, and steady-state peak and trough concentrations, and AUC are proportional to dosage.

Five studies with 945 patients treated with everolimus (lung, renal and neuroendocrine tumors) were included in a meta-analysis by Ravaud et al. where the mean everolimus Cminss was 15.65 ng/ml (90%CI 14,79–16.55 ng/ml) [17]. Better response and major reduction in tumor size were observed with a twofold increase in Cminss. In conclusion, Cminss ≥ 10 ng/ml could be used as target value for optimal response, while Cminss > 26.3 ng/ml was associated with a fourfold increased risk of toxicity compared to Cminss < 26.3 ng/ml. Only 45% of patients with neuroendocrine tumors achieved optimal everolimus plasmatic concentrations (10-30 ng/ml), while in lung and renal cancer were 55.2% and 62.9%, respectively. Strong CYP3A4 inhibitors increased everolimus Cminss by 10%, while CYP3A4 inducers decreased Cminss by 7%. In another study by Hirabatake et al. median PFS was 13.7 months (1.7–55.8 months) and 50% of breast cancer patients treated with everolimus showed Cminss below 10 ng/ml. PFS was significantly longer in the 10-20 ng/ml group (*p* = 0.0078) and the median of Cminss in patients with dose-limiting toxicities was 19 ng/ml (11.3–64.6 ng/ml) [18].

**Fig. 3:**
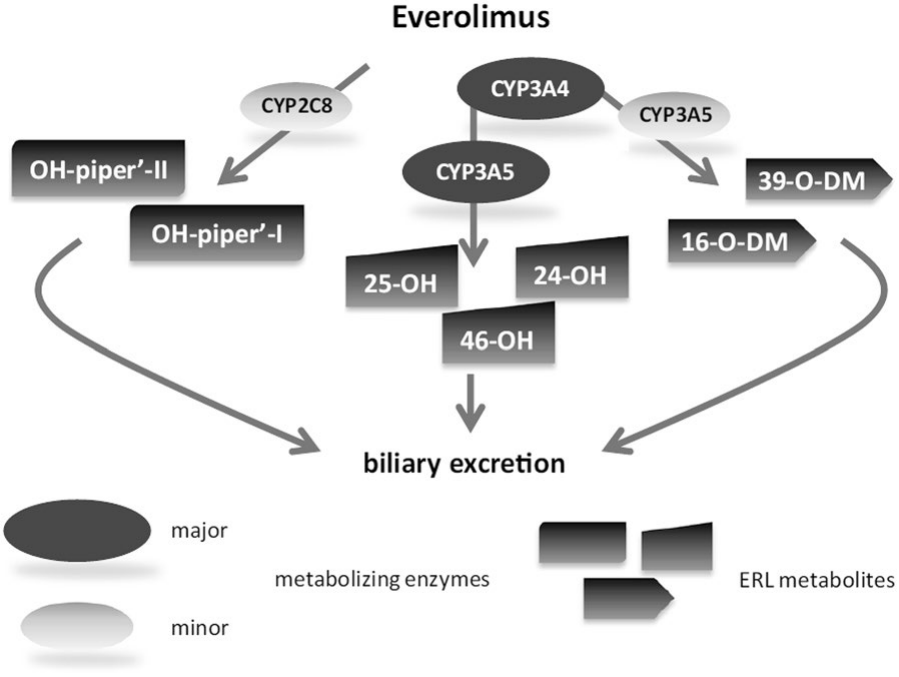
Everolimus metabolism in human liver

Inter-individual pharmacokinetic variability of everolimus can be explained by different activities of the drug efflux pump P-glycoprotein and of metabolism by cytochrome P450 (CYP) 3A4, 3A5 and 2C8 [13]. The critical role of the CYP3A4 system for everolimus biotransformation leads to drug–drug interactions with other drugs metabolied by this cytochrome system and could affect its efficacy or toxicity. Six main metabolites have been detected, but with 100 times less activity than parent drug (Fig. 3).

Two single-sequences, crossover studies with verapamil and erythromycin have quantified the influence of both drugs on the pharmacokinetics of everolimus.

In a study in 16 healthy subjects reported by Kovarik et al., verapamil (a relatively potent inhibitor of P-glycoprotein, and a moderate inhibitor of CYP3A4) administered as 80 mg three times daily, during 6 days, was added to a single 2 mg dose of everolimus [19]. During verapamil co-administration, everolimus Cmax increased 2.3-fold (90% CI 1.9,2.7) and AUC increased 3.5-fold (90% CI 3.1, 3.9). In the other study by Kovarik et al., erythromycin 500 mg (a CYP3A inhibitor) administered TID, for 9 days and a single 2-mg dose of everolimus were co-administered [20]. Everolimus *C *_max_ was raised up 2.0-fold (90% CI 1.8–2.3) and AUC was increased 4.4-fold (90% CI 3.5–5.4) during erythromycin co-administration.

A single drug–drug interaction between verapamil, clarithromycin and voriconazole with everolimus [21–23] has been reported in three case reports. Verapamil, clarithromycin, and voriconazole are known inhibitors of the CYP3A4 and their administration increased plasma concentration of everolimus (40 ng/ml –110 ng/ml) and its toxicities like mucositis, acute kidney injury, proteinuria or nephrotic syndrome. An interaction between fenofibrate and everolimus has been also reported. As CYP3A4 inducer, fenofibrate decreased the plasma concentration of everolimus (from 10.1 ng/ml to 4.2 ng/ml) [24].

To our knowledge, this is the first case report in the literature with two major interactions (carbamazepine and phenytoin) which can affect everolimus metabolism through strong induction of CYP3A4. First TDM Cminss was 3.7 ng/ml and everolimus dose was increase from 10 mg QD to 10mb BID to achieve optimal Cminss (> 10 ng/ml). Two months later, our patient finally achieved an optimal Cminss with a stabilization disease and PFS of 3.7 months.

The study by Verheijen et al. [25] investigated the pharmacokinetic optimization of everolimus dosing in oncology. The study was conducted as a crossover trial in 10 patients, and compared everolimus 10 mg once daily with 5 mg twice daily. The results of the study showed that the twice daily dosage regimen significantly decreased the maximum concentration (Cmax) of everolimus from 61.5 ng/ml to 40.3 ng/ml (*p* = 0.013), and significantly increased the minimum steady-state concentration (Cminss) from 9.6 ng/ml to 13.7 ng/ml (*p* = 0.018). However, there were no significant differences in the AUC between the two dosing regimens (435 ng*h/ml vs 436 ng*h/ml) (*p* = 0.952). These findings suggest that a twice daily dosing regimen of everolimus may be more effective in optimizing drug levels in oncology patients, compared to a once daily dosing regime.

In METEOR trial median PFS was 3.8 months when everolimus was used in renal cancer patients as second or third-line therapy, 73% and 27%, respectively [26]. In a perspective subanalysis in RECORD-1 trial, median PFS dropped from 5.4 months to 4 months when everolimus was used as third line in renal cancer patients who where previously treated with two anti-VEGFR tyrosine kinase inhibitor [27].

The limitations of this paper are only a case report and our findings cannot be generalized to larger groups of patients. TDM prospective studies are needed, specially in patients with different risk factors that may affect pharmacokinetic of everolimus. Nowadays the use of everolimus in metastatic RCC has recently decreased due to development of other TKIs and ICI, cabozantinib and nivolumab. The evidence about two new molecules, cabozantinib and nivolumab, successfully tested head-to-head with everolimus in recently published Phase III trials, will determine the shift of the drug to the third-line setting and subsequent lines of treatment. Promising data for its association with lenvatinib probably support the opportunity of everolimus to still remain in second-line setting for RCC treatment [28].

## Conclusion

Everolimus is metabolized by CYP3A4 and P-glycoprotein, and drug interactions at these levels can affect its blood concentrations, potentially leading to underexposure/overexposure and poor treatment outcomes in oncological patients. Major drug interactions are generally not allowed in clinical trials but in clinical practice TDM of everolimus could be essential in cancer patients receiving several concomitant medications or have other risk factors that may affect drug metabolism and elimination. Collaborative efforts between healthcare providers, can help to identify potential drug interactions and optimize the use of everolimus to achieve Cminss over 10 ng/ml. To realize the full potential of personalized medicine, we should treat each patient with the correct drug and its optimal dose.

## Data Availability

Everolimus treatment data were obtained from electronic prescription software. Medical and analytic data were obtained from electronic medical history.
